# Infective Endocarditis as a complication of COVID-19 infection; A case report and review of literature

**DOI:** 10.34172/jcvtr.2023.31618

**Published:** 2023-06-29

**Authors:** Khadije Mohammadi, Parya Soltani, Nazanin Davari

**Affiliations:** ^1^Cardiovascular Research Center, Kerman University of Medical Sciences, Kerman, Iran; ^2^Department of Radiology, Kerman University of Medical Sciences, Kerman, Iran

**Keywords:** COVID-19, Infective Endocarditis, Tricuspid Valve, Case Report

## Abstract

COVID-19 has been known to induce systemic inflammation and hyper coagulate state leading to different complications. Cardiovascular complications are one of the most important among complications following COVID-19 infection. A 57 years old woman with past medical history of COVID-19 infection about two months ago came to our hospital with presentation of fever and dyspnea. During workup, tricuspid valve infection associated with pulmonary septic emboli was diagnosed without any obvious risk factor for infective endocarditis. It seems that COVID-19 infection may increase the rate of endocarditis in patients with or without risk factors of endocarditis.

## Introduction

Coronavirus disease (COVID-19) originated at Wuhan, China; but very soon, spread worldwide and became a pandemic; concerning many countries in the world.^1^ It may cause a wide range of sign and symptoms.^1^ Most common presentations of this disease include fever, dyspnea, weakness, cough, myalgia, gastrointestinal upsets, also pneumonia, respiratory distress syndrome, renal and cardiac involvement and even death in more severe cases who need hospitalization.^1^ Considerable concerns risen following cardiovascular involvements of the COVID-19 infection. So that acute myocardial injury with high-sensitive troponin I elevation had been reported in 12% of the patients by Huang et al.^1^ Furthermore, in another study, in addition to 7.2% of acute myocardial injury, 16.7% of arrhythmias had been reported among 138 hospitalized patients with COVID-19 infection.^2^

Despite improvements in health care, Infective endocarditis (IE) remained a serious disease. Risk factors for IE can be classified in two groups, the first is transient bacteremia due to surgical procedures in the oral cavity, intravenous catheters, intravenous drug abuse or infections of skin, lungs, intestine, urinary tract and tooth; the second is an abnormal valve structure in context of congenital heart disease, degenerative disease, or previous cardiac surgery.^3^ This study aims to report a case of infective endocarditis following COVID-19 infection without any obvious risk factor.

## Description of the case

 The patient was a 57 years old woman, with chief complaints of fever, lethargy, fatigue and dyspnea from 10 days ago. She had no history of chest pain, nausea, orthopnea, or limb edema. In initial evaluation, she was ill and her vital signs were as follow: T: 38.5°C, Pulse Rate: 95 beats per minute, Respiratory Rate: 23/minute, Blood Pressure: 145/85mmHg and O2saturation: 90% by nasal cannula. In her physical examination, lymphadenopathy was not detected; there was no evidence of arthritis, conjunctivitis, cellulitis or visible skin lesion. In chest examination, lung sounds were decreased in right lower hemi thorax and also a whole systolic murmur was heard in left mid sternal border. No peripheral edema was noted.

**Figure 1:**
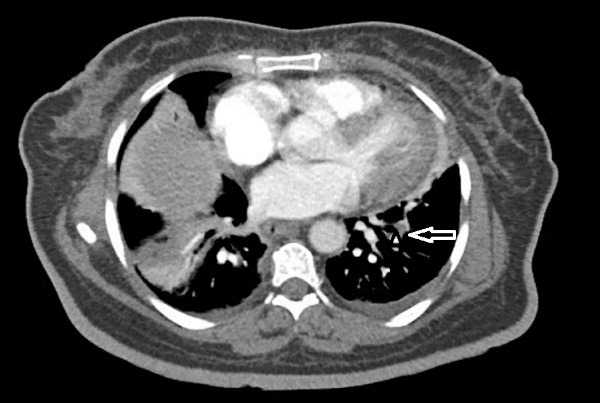
chest CT angiography that showed filling defects in sub segmental branches of basal segment of both pulmonary arteries (left side here) in favor of pulmonary emboli associated with sub segmental collapse consolidation in base of both lungs

She had a history of COVID-19 infection two month ago, with presentation of myalgia, fever and cough confirmed by positive nasopharyngeal swab test Polymerase Chain Reaction (PCR). Therefore, received supportive care at home quarantine for two weeks and didn’t experienced dyspnea or hypoxemia. In her chest Computed Tomography (CT) performed at that time, there was just faint patchy ground glass opacity in lingula and left lower lobe that could be due to COVID-19 infection. Her symptoms improved but 2 weeks after recovery, she developed acute unset of dyspnea which pulmonary thromboembolism was diagnosed in her work up (Figure 1, Supplementary Files, Video 1-3). She underwent anticoagulation therapy and was relatively good until 10 days before, which became febrile, associated with lethargy and loss of appetite and in outpatient visit. Then, antibiotic was prescribed for her but the fever persisted and she developed dyspnea. She also had a history of Diabetes Mellitus (DM), Hypertension (HTN) and hypothyroidism being on oral medication.

 According to the patient’s history, we considered complications of COVID-19 infection including cardiac involvement or pneumonia that complicating the pulmonary emboli.

**Figure 2:**
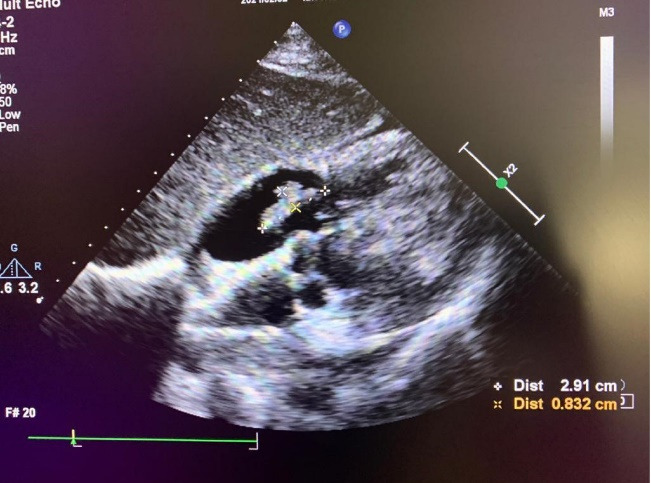
subcostal view of transthoracic echocardiography that showed large(2.9*0.8cm) mobile mass on atrial side of tricuspid valve, suggestive of vegetation

The results of initial laboratory tests showed leukocytosis (White Blood Cells: 18000 per microliter) with 82% neutrophils, Erythrocyte Sedimentation Rate (ESR):42mm/hour and C-Reactive Protein (CRP) was elevated. The blood cultures were also taken from two different sites and were negative. COVID-19 PCR test was done and was negative. Electrocardiogram (ECG) revealed sinus tachycardia, left axis deviation and evidence of Left Ventricular Hypertrophy (LVH). In echocardiography, left ventricular Ejection Fraction was normal with mild LVH with no wall motion abnormality. But a large (2.9 *0.8cm) mobile heterogeneous echo density was seen on atrial side of Tricuspid Valve (TV) that resulted in destruction of TV with severe Tricuspid Regurgitation (TR) in favor of vegetation. (Figure 2, Supplementary File, Video 4).

**Figure 3:**
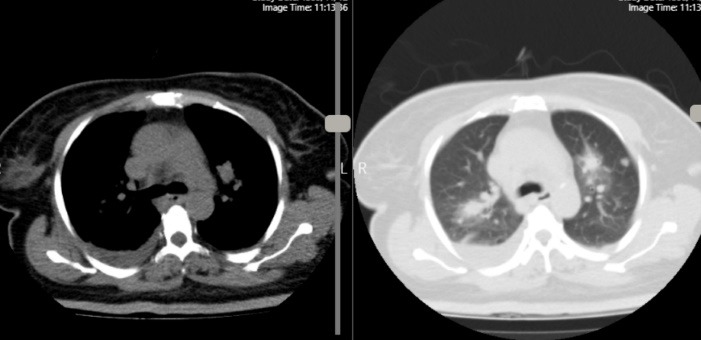
Chest CT revealed scattered nodules in both lungs with several consolidations and peripheral ground glass opacity in both upper and lower lobs of lung, suggestive of septic emboli

Chest CT revealed scattered nodules in both lungs with several consolidation and peripheral ground glass opacities (halo sign) in both upper and lower lobs of the lung suggestive of septic emboli. In addition, sub segmental collapse consolidation in base of Right Lower Lobe and mild pleural effusion in right side were seen. (Figure 3, Supplementary Files, Video 5, 6).

 According to modified Duke Criteria for infective endocarditis, the patient had one major and two minor criteria for IE. So the patient underwent experimental treatment of infective endocarditis with Meropenem and Vancomycine but despite anti-biotic therapy, the patient’s fever persisted. In lab data, WBC count of 2100 and ESR level of 81 were found. She underwent echocardiography again and no change in size of vegetation or no evidence of abscess formation was seen. Considering fever and vegetation size, the patient was scheduled for surgical excision of vegetation on TV with valve replacement. Intraoperative observation reported large vegetation associated with destructed valve; so it was replaced by a mechanical bileaflet prosthetic valve.

**Figure 4:**
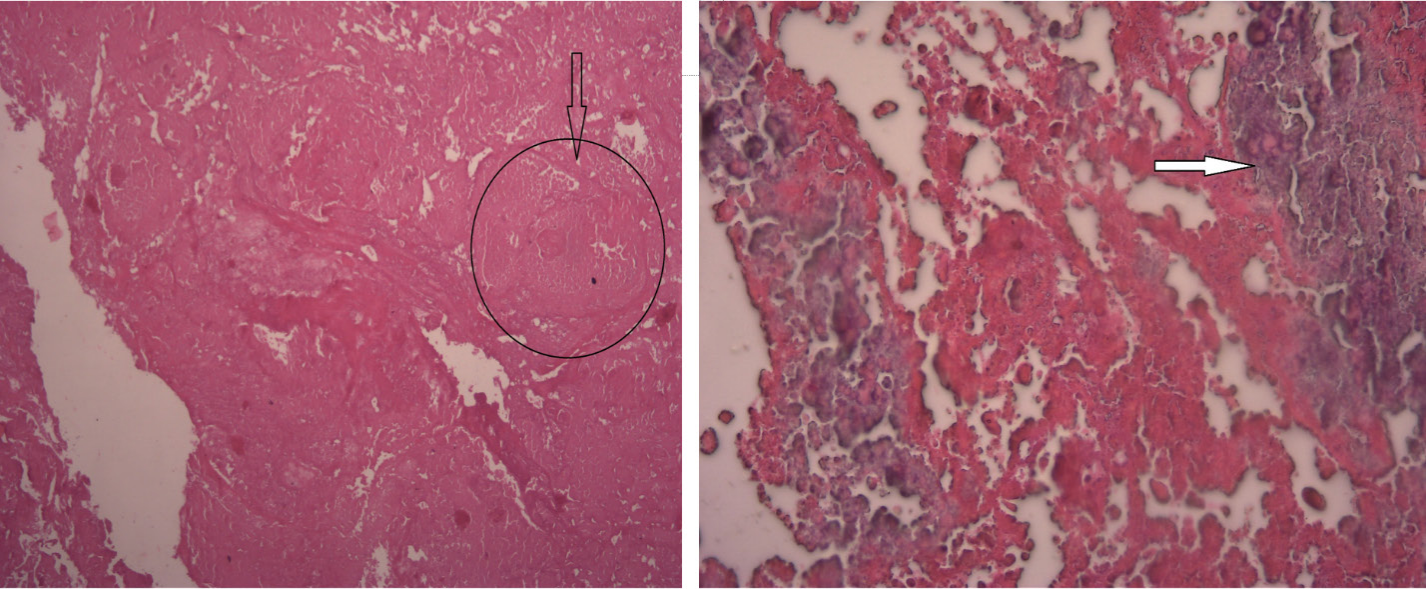
left: H and E, × 100, Endocardial tissue with necrosis and thrombose of blood vessel (Black arrow). Right: H and E, × 400, Endocardial tissue with necrosis, calcification and edema (white arrow)

The native valve tissue specimen was sent for pathologic examination and showed hyalinization, congestion and calcification with extensive necrosis, hemorrhage and foci of inflammatory cells infiltration. These microscopic findings were indicative of endocarditis. (Figure 4)

 Two days after surgery, the patient developed bradycardia and the ECG showed Complete Heart Block (CHB). Then, a temporary pace maker was implanted, soon replaced by a permanent epicardial one. Post-op transthoracic echocardiography showed good function of tricuspid prosthetic valve with no residual vegetation. The patient was discharged home after completion of antibiotic course and was uneventful in her follow up.

## Discussion

Following the outbreak of COVID-19 disease, its adverse effects were also commonly reported. Its cardio-vascular complications including acute coronary syndrome, atrial fibrillation, ventricular arrhythmia, myocarditis, pericarditis, hyper coagulate state and pulmonary thrombo-embolism, are one of the most important complications of COVID-19 infection. Although in some centers, an increased incidence of endocarditis has been reported during first months of COVID-19 pandemic ^4^ due to immunosuppressive therapies, central venous or urinary catheterization; some others showed decreased rate of endocarditis in this period.^5^

Escolà-Vergé et al examined the incidence of endocarditis between 2019 and 2 months of 2020 (in the early months of outbreak of COVID-19). They concluded that the reduced rate of IE in 2020 compare to 2019, may be related to instruction to stay at home, peoples fear of infection in medical facilities and avoiding medical cares in pandemic, overlap between symptoms of endocarditis and COVID infection and the prescription of oral antibiotics without further examination.^6^ However, their study was performed at the beginning of the covid-19 pandemic and over time, more cases of endocarditis were reported.

Currently, there is several case reports of endocarditis associated with COVID infection. In May 2020, Amir et al reported the first case of concomitant COVID infection with infective endocarditis that involving mitral valve in a patient with Rheumatic Heart Disease.^7^ About the aortic valve infection associated with COVID infection, we found 4 case reports during COVID pandemic, one in prosthetic aortic valve^8^ and the others in native valves.^9-11^ Tricuspid valve infection is relatively uncommon and usually occurs in patient with risk factors. Until now two cases of TV endocarditis were reported in context of COVID infection. One of them was in a patient with history of trauma that presented with severe respiratory distress and positive test for SARS-CoV-2 and another one was in a intubated COVID patient with central venous catheter.^12,13^

 The important point about these cases is that in most of them, there were several risk factors for bacteremia and IE like central venous lines, urinary catheters, mechanical ventilation, RHD, and mechanical valve. Moreover, many of them had been received immunosuppressive therapies for COVID infection; although it’s not clear that these treatments increase risk of endocarditis or not.

But in the present case with TV endocarditis, there is not any classic risk factor for IE. In her past medical history, she only had a history of mild COVID-19 infection that complicated with pulmonary emboli. Therefore, she did not receive immunosuppressive therapy in her disease course nor central venous or urinary catheter was fixed for her. We justified negative blood culture as a result of outpatient antibiotic treatment. In addition, late presentation of endocarditis is very important in this case. All mentioned IE cases in previous part were developed during COVID 19 disease course but in our case IE was diagnosed 2 months after COVID 19. Similarly, Kumanayaka et al reported a case of mitral valve endocarditis one month after COVID infection in a patient without IE risk factor rather than history of coronavirus 19 and treatment with dexamethasone.^3^ Alizadehasl et al also reported a case of prosthetic mitral IE 3weeks after COVID infection.^14^ So, it seems that the role of COVID 19 could not be ruled out as a predisposing factor for IE either early or late. Even though many of reported cases had other risk factors, but some others like our case didn’t have any obvious risk factor for IE other than COVID infection and this hypothesis is highlighted by the study of Aikawa et al who reported evidence of late onset non-bacterial endocarditis following COVID 19 infection in imaging and biopsy.^15^ It seems that systemic inflammation and hyper coagulate state induced by COVID-19 infection, may be responsible for its complications including IE.

## Conclusion

 It seems that covid-19 infection may increase the rate of endocarditis in patients with or without risk factors of endocarditis. So, in patients with Covid-19, if the fever persists or evidence of septic embolism are seen in imaging, we should consider the complications of the disease, especially endocarditis. In addition, we should be careful about selecting patients for hospitalization, implantation of venous lines or urinary catheters and immunosuppressive treatment to reduce rate of endocarditis.

## Acknowledgments

 This is a scientific article in the field of medical science. I would like to thank research committee, Dr.Soltani and Dr.Mohammadi for their insightful comments and encouragement.

## Competing Interests

 The authors declare that they have no competing interests.

## Ethical Approval

 The study was approved in the research ethics committee of Kerman University of Medical Sciences. The patient signed the written informed consent for the publication of this case report study including all of the images, available for reviewers. The patient was assured that all informations will remain anonymous and confidential.

## Funding

 None to declare.

## Supplementary Files


Supplementary file 1: Video 1.

Supplementary file 2: Video 2.

Supplementary file 3: Video 3.

Supplementary file 4: Video 4.

Supplementary file 5: Video 5.

Supplementary file 6: Video 6.
